# Geographic disparities and predictors of vaccination exemptions in Florida: a retrospective study

**DOI:** 10.7717/peerj.12973

**Published:** 2022-02-22

**Authors:** Corinne B. Tandy, Agricola Odoi

**Affiliations:** Biomedical and Diagnostic Sciences, University of Tennessee, Knoxville, TN, United States

**Keywords:** Vaccination, Vaccination exemptions, Geographic information systems, GIS, Geographic disparities, Socio-demographic predictors, Socioeconomic predictors, Demographic predictors, Negative binomial regression, Florida

## Abstract

**Background:**

In the United States, state-level policies requiring vaccination of school-going children constitute a critical strategy for improving vaccination coverage. However, policies allowing vaccination exemptions have also been implemented and contribute to reductions in vaccination coverage and potential increases in the burden of vaccine-preventable diseases. Understanding the geographic disparities in the distribution of vaccination exemptions and identifying high risk areas is necessary for guiding resource allocation and public health control strategies. This study investigated geographic disparities in vaccination exemptions as well as socioeconomic and demographic predictors of vaccination exemptions in Florida.

**Methods:**

Vaccination exemption data were obtained from the Florida Department of Health’s Florida HealthCHARTS web interface. Spatial patterns in geographic distribution of total and non-medical vaccination exemptions were assessed using county-level choropleth maps. Negative binomial models were used to identify significant predictors of county-level risks of both total and non-medical vaccination exemptions.

**Results:**

Total exemptions varied from 0 to 30.2 per 10,000 people. Nine counties had exemption risks in the top two classes (10.4–15.9 and 15.9–30.2 exemptions per 10,000 people). These counties were distributed in five distinct areas: Western Panhandle, central northern area, central, South-eastern coastal area, and the southern coastal border of the state. Non-medical exemptions varied from 0 to 10.4 per 10,000 people. Fifteen counties had exemption risks in the top two classes (3.7–5.6 and 5.6–10.4 exemptions per 10,000 people), and were located in six distinct areas: Western and Central Panhandle, Northeastern, Central-eastern coastal area, Central-western coastal area, and the South-western coastal border of the state. Predictors of high risk of total vaccination exemptions were high density of primary care providers (*p* < 0.001), high median income (*p* = 0.001), high percentage of Hispanic population (*p* = 0.046), and low percentage of population with a college education (*p* = 0.013). A predictor of high risk of non-medical vaccination exemptions was high percentage of White population (*p* = 0.045). However, predictors of low risks of non-medical exemptions were high percentages of population: living in rural areas (*p* = 0.023), with college education (*p* = 0.013), with high school education (*p* = 0.009), and with less than high school education (*p* < 0.001).

**Conclusions:**

There is evidence of county-level geographic disparities in both total and non-medical vaccination exemption risks in Florida. These disparities are explained by differences in county-level socioeconomic and demographic factors. Study findings are important in guiding resource allocation for health planning aimed at improving vaccination rates and reducing incidence of vaccine-preventable diseases.

## Introduction

The United States and Europe are experiencing an increase in incidence of vaccine-preventable diseases which has been attributed to anti-vaccine movements (Hotez, 2019). As a result, the United States has experienced multiple outbreaks of vaccine-preventable diseases such as measles and pertussis among mostly unvaccinated persons (Sanyaolu et al., 2019). The resurgence of vaccine preventable diseases has been, at least partially, attributed to decreased vaccination coverage.

There is evidence that vaccination coverage varies by geographic region. In the United States, state-level policies requiring vaccination of school-going children have been identified as important strategies for improving vaccination coverage (Omer et al., 2009). On the other hand, policies allowing vaccination exemptions, particularly philosophical and religious exemptions, have been implicated in contributing to reductions in vaccination coverage and potential increases in the burden of vaccine-preventable diseases (Blank, Caplan & Constable, 2013). Unfortunately, vaccination exemption policies are not consistent across the country and vary widely from state to state. Currently, all 50 US states and Washington DC allow medical exemptions for school-going children while 44 states and Washington DC allow religious exemptions, and 15 allow philosophical exemptions (National Conference of State Legislatures, 2022). Across the country, only California, Maine, New York, Mississippi, and West Virginia do not allow non-medical exemptions (Constable, Blank & Caplan, 2014; Williams et al., 2019; National Conference of State Legislatures, 2021). Since states vary widely in the rates of accommodations of religious or philosophical objections to vaccination (Omer et al., 2006, 2008; Atwell et al., 2013; Constable, Blank & Caplan, 2014), the ease of getting vaccination exemptions varies widely. As a result, states having more strict or arduous exemption processes have lower rates of vaccination exemptions (Omer et al., 2006, 2008; Atwell et al., 2013; Constable, Blank & Caplan, 2014). The rigor of the approval process for religious exemptions also varies significantly across the country and often only requires a parent/guardian’s signature (Blank, Caplan & Constable, 2013).

The role of vaccine exemptions in the transmission of vaccine-preventable diseases, particularly measles and pertussis, has been of particular interest as incidence rates of vaccine-preventable diseases have increased. Omer et al. (2006) reported that philosophical/personal belief exemptions were associated with increased pertussis incidence (Incidence Risk Ratio [IRR] = 1.48) between 2000 and 2004 (Omer et al., 2006). Similarly, Atwell et al. (2013) geographic analysis of nonmedical exemptions and pertussis risk in California reported that clusters of non-medical exemptions at the census tract level were associated with higher pertussis risk (IRR = 1.20). An understanding of the predictors of vaccination exemptions may be useful in guiding public health policy decisions and efforts geared towards improving vaccination coverage.

Non-medical reasons for vaccination exemptions may be closely related to population socioeconomic and demographic characteristics that, if identified, would guide efforts to reduce these non-medical exemptions, improve vaccination coverage, and better control vaccine-preventable diseases. Therefore, the objectives of this study were to identify: (a) geographic disparities in prevalence of vaccination exemptions; (b) socioeconomic and demographic predictors of vaccination exemptions among school-going children in Florida.

## Materials and Methods

### Study area

**Figure 1:**
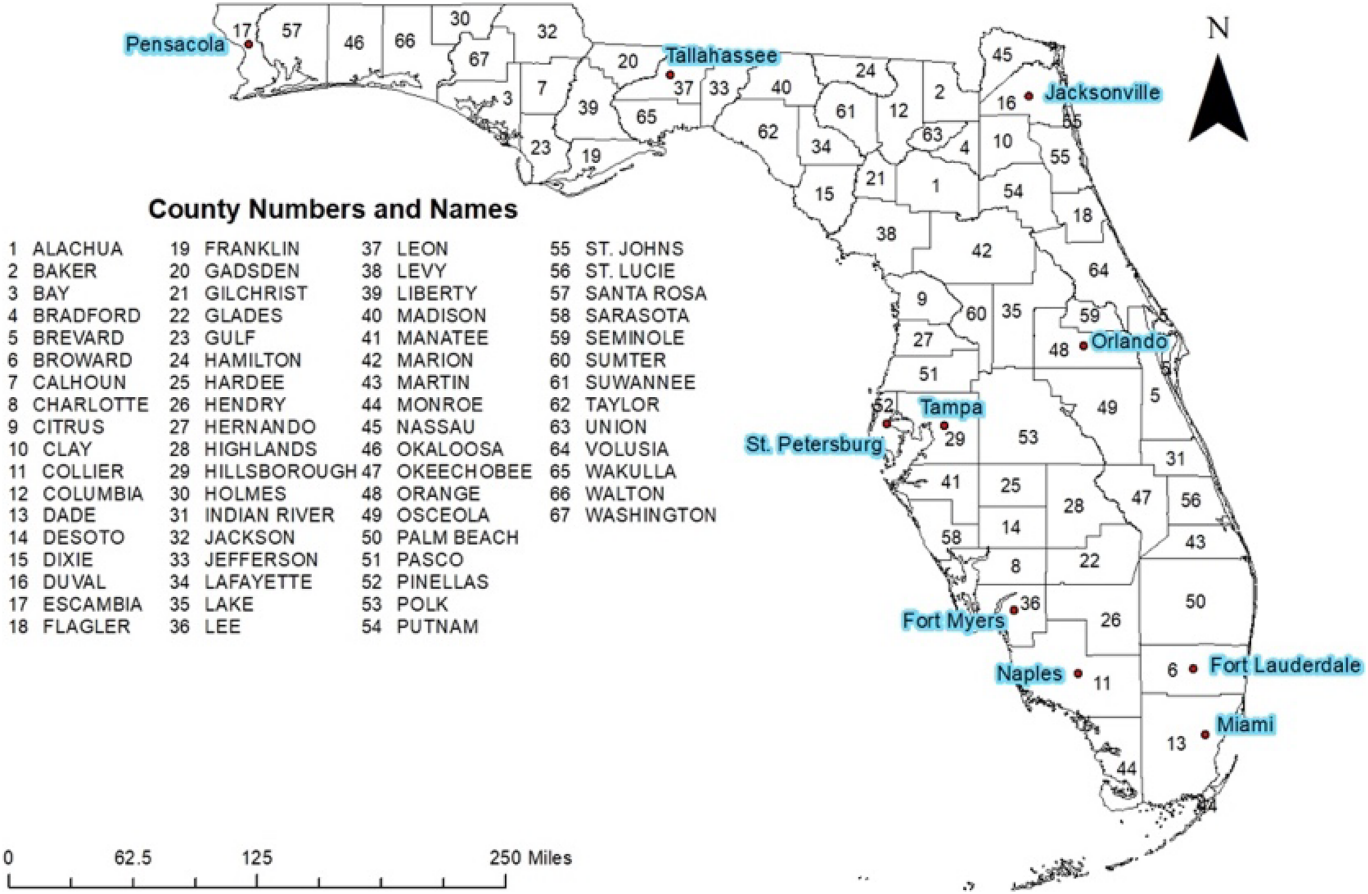
Geographic distribution of counties and major cities of Florida.

This retrospective study was conducted in Florida, which consists of 67 counties, both rural and urban (Fig. 1). Miami-Dade County is the most urban and most populated with approximately 2.7 million residents while Liberty County is the most rural and least populated county with approximately 8,300 residents (US Census Bureau, 2018). Florida population is comprised of 53.2% non-Hispanic White, 26.4% Hispanic, 3% Asian, and 16.9% Black or African American. The remaining 0.5% of the population are Native Hawaiian or Other Pacific Islander or two or more races (US Census Bureau, 2020). The population is 51.1% female and 48.9% male and has the following age distribution: <5 years (5.3%), 6–18 years (19.7%), 19–64 (54.1%), and 65 years and older (20.9%) (US Census Bureau, 2020).

### Data sources and cartographic displays

County-level vaccination exemption and population data for 2014 were downloaded from Florida Health CHARTS (Florida Department of Health, 2020). Cartographic boundary files, used for creating maps, were obtained from the United States Census Bureau’s TIGER files (United States Census Bureau, 2019) and the State of Florida Geographic Data Portal (Southwest Florida Water Management District, 2019). Vaccination exemptions per 10,000 population at the county level were calculated and displayed in choropleth maps using ArcGIS (ArcGIS desktop version 10.7; Esri, Redlands, CA, USA). Critical intervals of the choropleth maps were determined using Jenk’s optimization classification scheme.

### Predictors of total and non-medical county-level vaccination exemption risks

**Figure 2:**
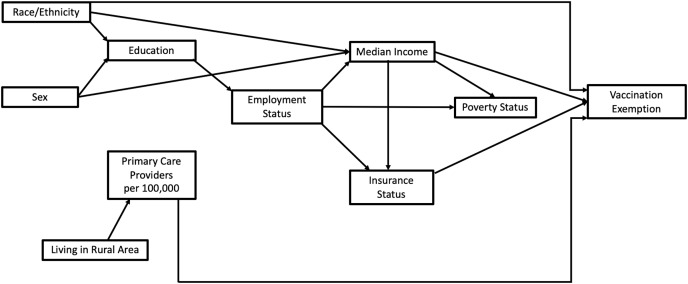
Conceptual model of sociodemographic variables considered for analysis of vaccination exemptions in Florida, 2014.

A conceptual model was used to identify potential county-level predictors of vaccination exemption risks (Fig. 2). Based on the conceptual model, a total of 16 potential predictors were assessed for potential associations with either total and non-medical vaccination exemption risks (Table 1). Predictors of total and non-medical county-level vaccine exemption risks were investigated using two sets of Poisson models for the two outcomes, total and non-medical vaccination exemptions. The first step in building the models involved fitting univariable models between each of the potential predictors and each of the outcome variables (number of total and non-medical vaccination exemptions) with population used as the offset. Univariable associations were assessed at a liberal *p*-value of 0.2 and variables with a *p* < 0.2 were considered for further investigation in step two. To minimize potential multicollinearity, two-way Spearman rank correlation coefficients were computed among variables that had *p* < 0.2. Only one of a pair of highly correlated variables (*i.e*., with r > 0.7) was retained for assessment in step 2 of the modeling process. The decision on the variable to retain was guided by biological and statistical considerations.

**Table 1:** Summary statistics of potential predictors of vaccination exemptions in Florida, 2014.

Variable	Median	1st quartile	3rd quartile
Less than high school education (%)	14.5	11.5	21.7
High school education (%)	34.3	28.9	37.5
College education (%)	18.6	11.4	26.7
White, non-Hispanic (%)	83.0	77.8	87.6
Black, non-Hispanic (%)	13.1	8.6	19.1
Hispanic (%)	8.65	5.29	18.1
Male (%)	49.4	48.6	53.3
Female (%)	50.6	46.7	51.4
Unemployed (%)	6.6	5.8	7.2
Uninsured (%)	18.1	15.6	21.1
Family below poverty line (%)	12.8	10.1	16.9
Individuals below poverty line (%)	17.7	14.3	22.5
PCP^1^ (per 10,000 population)	48.0	28.0	68.0
% Living in rural area	23.8	8.5	67.5
Rural County (*vs* Urban)	–	–	–
Scaled median income (Divided by $10,000)	43,063	36,907	48,483

**Note:**

1 Primary Care Providers.

The second step in the modeling process involved fitting multivariable Poisson model using backwards elimination with the predictor variables that had *p* < 0.2 in step 1 but this time using a critical *p*-value of 0.05. Confounding was assessed by examining whether the removal of a predictor variable resulted in a >20% change in the coefficients of any other variables in the model (Dohoo, Martin & Stryhn, 2012). Identified confounders were retained in the models regardless of their statistical significance. Overdispersion was assessed by comparing model deviance and degrees of freedom. The Poisson models showed evidence of overdispersion and, therefore, negative binomial regression models were fit to the data using the same process outlined above for the Poisson model. All statistical analyses were performed in STATA version 16.1 (StataCorp, 2019). Overall goodness-of-fit of the final negative binomial models were assessed using Deviance and Pearson Chi-square goodness-of-fit tests. Spatial autocorrelation of the deviance, Pearson and Anscombe residuals of the negative binomial models were assessed using Moran’s I implemented in GeoDa (Anselin, 2019). Statistical significance of the computed Moran’s I values were assessed using permutations approach based on 999 permutations.

### Ethical statement

This study was reviewed by the University of Tennessee, Knoxville Institutional Review Board (IRB Number: UTK IRB-20-05957). The board determined that it did not involve human subjects and, therefore, did not require IRB oversight.

## Results

The largest number of exemptions was in Miami-Dade County (4,157) while the lowest was in Lafayette county which had no exemptions during the study period. The highest percentages of exemptions were seen in Seminole (4.8%), Miami-Dade (2.6%), Columbia (2.6%), Palm Beath (2.4%), Sarasota (2.3%) and St. John’s (2.0%) counties.

### Geographic patterns of vaccination exemptions

**Figure 3:**
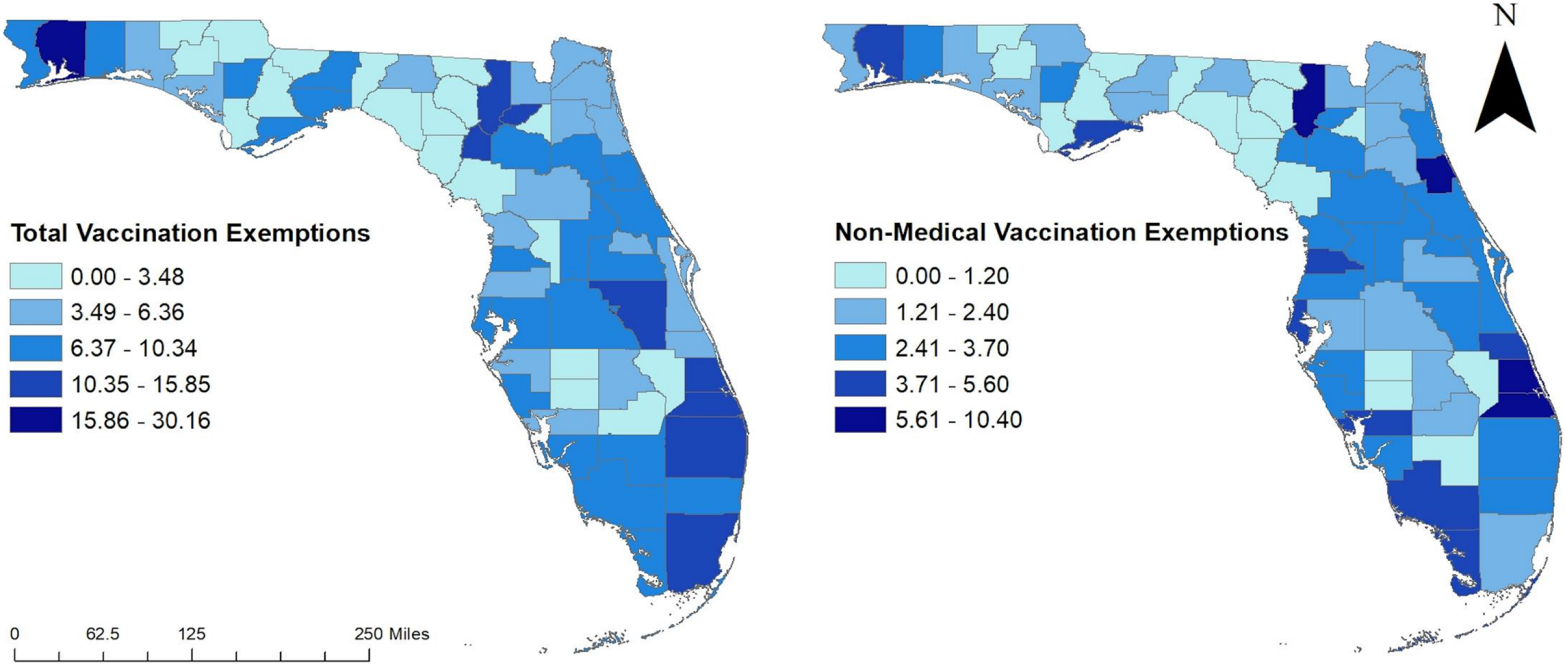
Total and non-medical vaccination exemptions per 10,000 population in Florida, 2014.

Total exemptions varied from 0 to 30.16 per 10,000 population (Fig. 3). Nine counties had exemption risks in the top two classes (10.35–15.85 and 15.86–30.16 exemptions per 10,000 population) (Fig. 3). These counties were distributed in five distinct areas of the state: Western Panhandle (Santa Rosa County), central northern area (Columbia, Union, and Gilchrist counties, central (Osceola County)), South-eastern coastal area (Palm Beach, Martin, and St. Lucie counties), and the southern coastal border of the state (Miami-Dade County) (Figs. 1 and 3). Areas with the lowest exemptions (0–3.48 per 10,000 population) were primarily seen in the central portion of the state (Hardee, Desoto, Glades, Okeechobee counties), in the central panhandle (Holmes, Washington, Jackson, Gadsden, Liberty, and Gulf counties), and along north-western coastal and northern parts of the state (Jefferson, Taylor, Lafayette, Dixie, Levy, Suwannee, and Hamilton counties). Two other counties in the northern central part of the state also had low risks of total exemptions (Bradford and Sumter counties).

Non-medical exemptions varied from 0 to 10.40 per 10,000 population (Fig. 3). Fifteen counties had exemption risks in the top two classes (3.71–5.60 and 5.61–10.40 exemptions per 10,000 population) (Fig. 3). These counties were distributed in six distinct areas of the state: Western Panhandle (Santa Rosa and Okaloosa Counties), Central Panhandle (Franklin County), Northeastern (Flagler and Columbia Counties), Central-eastern coastal area (Indian River, Brevard, Martin, and St. Lucie Counties), Central-western coastal area (Pinellas, Hernando, and Citrus Counties), and the South-western coastal border of the state (Monroe, Collier, and Charlotte Counties) (Figs. 1 and 3). Areas with the lowest non-medical exemptions (0–1.20 per 10,000 population) were primarily seen in the central portion of the state (Hardee, Desoto, Hendry, and Okeechobee Counties), in the central panhandle (Holmes, Washington, Gadsden, Liberty, and Gulf counties), and along the northern gulf and eastern panhandle area (Jefferson, Taylor, Dixie, Levy, Lafayette, Suwannee, and Hamilton counties). One other county in the northern central part of the state also had low medical exemptions (Bradford County).

### Predictors of total vaccination exemptions

Results of univariable and multivariable Poisson models investigating predictors of total exemptions are shown in Tables 2 and 3. Since the results of the multivariable Poisson model showed evidence of overdispersion (Deviance/df = 29.7; Pearson Chi-square/df = 34.02; Likelihood Ratio Test of overdispersion parameter = 0, *p* < 0.001), a negative binomial model was more appropriate for these data and hence the results of investigation of predictors of total vaccination exemptions focuses on the negative binomial model results (Table 4). Based on the results of this model, the risk of total vaccination exemptions tended to be higher in counties with more primary care providers per 10,000 population, higher median income, higher percentage of Hispanic population, and higher percentage of population with a college education than in counties with lower values of these predictors (Table 4). There was no evidence of lack of fit of the total exemptions negative binomial model based on the Deviance goodness-of-fit test (*p* = 0.169). Similarly, there was no evidence of spatial autocorrelation of the deviance residuals (Moran’s I = −0.067; *p* = 0.272), Pearson residuals (Moran’s I = −0.052; *p* = 0.353) or Anscombe residuals (Moran’s I = 0.026; *p* = 0.257).

**Table 2:** Results of univariable associations of potential predictors of total vaccination exemptions in Florida, 2014.

Variable	Risk ratio	Lower 95% CI^1^	Upper 95% CI^1^	*p*-value
Less than high school education (%)	1.036	1.032	1.039	<0.001
High school education (%)	0.968	0.964	0.972	<0.001
College education (%)	1.012	1.009	1.015	<0.001
White, non-Hispanic (%)	0.998	0.997	1.000	0.072
Black, non-Hispanic (%)	1.005	1.003	1.007	<0.001
Hispanic (%)	1.014	1.012	1.014	<0.001
Male (%)	0.954	0.941	0.966	<0.001
Female (%)	1.049	1.035	1.062	<0.001
Unemployed (%)	0.958	0.939	0.977	0.282
Uninsured (%)	1.059	1.056	1.062	<0.001
Family below poverty line (%)	1.047	1.042	1.052	<0.001
Individuals below poverty line (%)	1.024	1.019	1.028	<0.001
PCP^2^ (per 10,000 population)	1.007	1.006	1.008	<0.001
% Living in rural area	0.986	0.984	0.987	<0.001
Rural County (*vs* Urban)	0.523	0.477	0.573	<0.001
Scaled median income (Divided by $10,000)	1.011	0.983	1.039	0.454

**Notes:**

1 95% Confidence Interval.

2 Primary Care Providers.

**Table 3:** Results of the final poisson model showing significant predictors of total vaccination exemptions in Florida, 2014.

Variable	Risk ratio	Lower 95% CI^1^	Upper 95% CI^1^	*p*-value
PCP^2^ (per 10,000 population)	1.002	1.000	1.004	0.046
Rural (%)	0.997	0.995	0.999	0.041
Rural (*vs* Urban)	0.421	0.360	0.493	<0.001
White (%)	1.069	1.053	1.084	<0.001
Black (%)	1.063	1.045	1.080	<0.001
Uninsured (%)	1.039	1.032	1.047	<0.001
Male (%)	1.054	1.029	1.080	<0.001
Unemployed (%)	0.873	0.835	0.913	<0.001
Individuals below poverty (%)	1.0147	1.003	1.027	0.014
High school education (%)	0.919	0.901	0.936	<0.001
Scaled median income (Divided by $10,000)	1.101	1.013	1.198	0.023
College education (%)	0.948	0.937	0.960	<0.001

**Notes:**

1 95% Confidence Interval.

2 Primary Care Providers.

**Table 4:** Results of the final negative binomial model showing significant predictors of total vaccination exemptions in Florida, 2014.

Variable	Risk ratio	Lower 95% CI^1^	Upper 95% CI^1^	*p*-value
PCP^2^ (per 10,000 population)	1.017	1.008	1.027	<0.001
Scaled median income (Divided by $10,000)	1.523	1.181	1.964	0.001
Hispanic (%)	1.009	1.00	1.019	0.046
College education (%)	0.962	0.933	0.992	0.013

**Notes:**

1 95% Confidence Interval.

2 Primary Care Providers.

### Predictors of non-medical vaccination exemptions

Findings of the univariable and multivariable Poisson models used to investigate predictors of non-medical exemptions are shown in Tables 5 and 6. As was the case with the total exemptions model, there was evidence of over-dispersion (Deviance/df = 13.98; Pearson Chi-square/df = 16.28; Likelihood Ratio Test of over-dispersion parameter = 0, *p* < 0.001) of the final Poisson model (Table 6) used to investigate predictors of non-medical exemptions implying that the Poisson model was not appropriate for the investigation. Therefore, subsequent results presented are based on the final multivariable negative binomial model (Table 7). Based on this model, high risks of non-medical exemptions tended to occur in counties with higher percentage of white population. By contrast, lower risks of non-medical exemptions tended to occur in counties with higher percentages of the population: (a) living in rural areas, (b) with a college education, (c) having high school education, and (d) having less than a high school education. As for the total exemptions model, this model indicated no evidence of lack of fit based on the Deviance goodness-of-fit test (*p* = 0.153) although the Pearson goodness of fit test indicated that the model did not fit the data well (*p* = 0.003). There was also no evidence of spatial autocorrelation of the deviance residuals (Moran’s I = 0.011; *p* = 0.343), Pearson residuals (Moran’s I = 0.036; *p* = 0.205) or Anscombe residuals (Moran’s I = 0.011; *p* = 0.075).

**Table 5:** Results of univariable associations of potential predictors of non-medical vaccination exemptions in Florida, 2014.

Variable	Risk ratio	Lower 95% CI^1^	Upper 95% CI^1^	*p*-value
Less than high school education (%)	0.956	0.949	0.963	<0.001
High school education (%)	1.010	1.003	1.017	0.002
College education (%)	0.994	0.990	0.999	0.007
White, non-Hispanic (%)	1.010	1.007	1.013	<0.001
Black, non-Hispanic (%)	0.986	0.983	0.989	<0.001
Hispanic (%)	0.989	0.988	0.991	<0.001
Male (%)	1.032	1.014	1.049	<0.001
Female (%)	0.969	0.953	0.986	<0.001
Unemployed (%)	0.977	0.945	1.011	0.177
Uninsured (%)	0.969	0.964	0.976	<0.001
Family below poverty line (%)	0.938	0.929	0.947	<0.001
Individuals below poverty line (%)	0.948	0.939	0.955	<0.001
PCP^2^ (per 10,000 population)	0.996	0.995	0.998	<0.001
% Living in rural area	0.999	0.008	1.001	0.563
Rural County (*vs* Urban)	0.845	0.745	0.958	0.008
Scaled median income (Divided by $10,000)	1.229	1.173	1.285	<0.001

**Notes:**

1 95% Confidence Interval.

2 Primary Care Providers.

**Table 6:** Results of the final poisson model showing significant predictors of non-medical vaccination exemptions in Florida, 2014.

Variable	Risk ratio	Lower 95% CI^1^	Upper 95% CI^1^	*p*-value
Scaled median income (Divided by $10,000)	1.631	1.444	1.843	<0.001
Rural (%)	0.993	0.989	0.996	<0.001
Rural (*vs* Urban)	0.740	0.595	0.920	0.007
White (%)	1.053	1.023	1.084	<0.001
Black (%)	1.050	1.019	1.083	0.002
Hispanic (%)	0.988	0.982	0.993	<0.001
Male (%)	1.082	1.044	1.120	<0.001
Families below poverty (%)	0.904	0.861	0.949	<0.001
Individuals below poverty (%)	1.119	1.081	1.157	<0.001
Unemployed (%)	1.082	1.018	0.913	0.012
Uninsured (%)	1.082	1.062	1.027	<0.001
Less than high school (%)	0.905	0.885	0.926	<0.001
High school education (%)	0.918	0.892	0.944	<0.001
College education (%)	0.897	0.879	0.916	<0.001

**Note:**

1 95% Confidence Interval.

**Table 7:** Results of the final negative binomial model showing significant predictors of non-medical vaccination exemptions in Florida, 2014.

Variable	Risk ratio	Lower 95% CI^1^	Upper 95% CI^1^	*p*-value
Rural (%)	0.992	0.986	0.999	0.023
White (%)	1.016	1.000	1.032	0.045
College education (%)	0.897	0.846	0.951	<0.001
High school education (%)	0.898	0.829	0.973	0.009
Less than high school (%)	0.908	0.874	0.944	<0.001

**Note:**

1 95% Confidence Interval.

## Discussion

This study identified county-level geographic disparities and sociodemographic predictors of both total and non-medical vaccination exemptions in Florida in 2014. Study findings provide information that is useful for guiding public health programs and policy.

### Geographic disparities in vaccination exemptions

#### Total exemptions

The identification of county-level geographic disparities of total vaccination exemptions in this study is consistent with findings from other studies that investigated geographic patterns of vaccination exemptions and vaccine-preventable diseases (Omer et al., 2006; Aloe, Kulldorff & Bloom, 2017). An examination of total vaccination exemptions in Florida between 2013 and 2019 reported variations of exemptions at the county level (Muller & Gwynn, 2021). The same study reported that, within Miami-Dade county, vaccination exemptions varied by school during the 2017–2018 school year (Muller & Gwynn, 2021). Another study which investigated clustering of vaccination exemptions by exemption type (medical, religious, and philosophical) at the school level in Michigan identified clusters for each exemption type, indicating that geographic differences occur by type of exemption (Mashinini et al., 2020). Medical exemptions clustered in the southeast and northwestern regions of the state, religious exemptions were clustered in the southeastern areas while philosophical exemptions clustered in the southeast. Another analysis of total vaccination exemptions in Ontario reported that total vaccination exemptions varied greatly by geographic area (Wilson et al., 2015). The same study reported that communities with higher vaccination exemption rates tended to report outbreaks of vaccine-preventable diseases in southwestern Ontario, including a 2005 rubella outbreak and a 2011 pertussis outbreak (Wilson et al., 2015).

#### Non-medical exemptions

The geographic disparities in non-medical vaccination exemptions observed at the county level in this study are consistent with findings from other studies that have reported that non-medical exemptions are often geographically clustered (Omer et al., 2006). This is a cause for concern as clustering of unvaccinated or under-vaccinated individuals increases the risk of outbreaks of vaccine-preventable diseases (Parker et al., 2006). A geospatial study of non-medical vaccine exemptions and pertussis outbreaks in the United States reported that geographic clusters of non-medical exemptions were associated with pertussis outbreaks in children under five and those aged 10–14 years (Aloe, Kulldorff & Bloom, 2017). Clusters of non-medical exemptions have also been identified in a school-level cluster analysis in Miami-Dade County in the 2017–2018 school year, during which wide variations between schools were reported (Muller & Gwynn, 2021). The study also reported that several schools had less than 90% vaccine compliance (Muller & Gwynn, 2021).

### Predictors of vaccination exemptions

#### Total exemptions

The observed association between higher median income and total vaccination exemptions in this study is inconsistent with reports by Quinn, Jamison & Freimuth (2020) from a study of public attitudes towards vaccination exemptions. The authors reported that adults with an annual income greater than $40,000 favored the idea of required vaccination and had less favorable attitudes towards exemptions (Quinn, Jamison & Freimuth, 2020). However, since the study by Quinn, Jamison & Freimuth (2020) reported individual-level attitudes towards vaccination, direct comparisons cannot be made with the findings of this county-level study and should be interpreted with caution.

The finding that higher percentage of Hispanic population was a predictor of higher risk of total exemptions in this study contrasts reports from an Arizona study which reported that Hispanic ethnicity was associated with higher vaccination acceptance and coverage (Birnbaum et al., 2013). Although the observed association between density of primary care providers and total vaccination exemptions in this study has not been reported elsewhere, there have been reports of association between density of primary care providers and non-medical exemptions (Walker & Rea, 2016).

#### Non-medical exemptions

The association between white race and higher risk of non-medical vaccination exemptions observed in this study is consistent with findings from several other studies that have consistently reported that White race was a predictor of both higher non-medical exemptions and vaccination hesitancy and refusals (Gust et al., 2005, 2008; Kennedy, Brown & Gust, 2005; Kennedy et al., 2011; Birnbaum et al., 2013; Richards et al., 2013; Yang et al., 2016; Morrison, Castro & Meyers, 2020). Yang et al. (2016) explored the relationship between sociodemographic factors and philosophical exemptions in California and reported that personal belief exemptions were higher in areas with higher percentages of white populations (Yang et al., 2016). Similarly, a study of Arizona school systems reported that schools with higher proportions of white students had the highest philosophical/personal belief vaccination exemptions (RR = 14.11) (Birnbaum et al., 2013).

Counties with higher percentages of people living in rural areas had lower risk of non-medical vaccination exemptions, a finding that is consistent with those from a study in California which reported lower exemption rates in rural areas (Yang et al., 2016). However, a longitudinal analysis of community factors associated with non-medical exemptions in California reported that schools in rural areas had higher non-medical exemption rates than those in urban areas (Richards et al., 2013). Since that study investigated exemptions at the census tract level, the differences in findings at different geographic levels implies that future investigations should consider assessing several geographic scales including counties, census tracts or school districts.

The finding that higher percentage of the population having a college degree were associated with lower risk of non-medical vaccination exemptions is consistent with findings from previous studies. For example, Yang et al. (2016) reported that higher education level was associated with a lower percentage of exemptions at school and regional geographic levels. The current study also identified that counties with higher percentage of the population having high school education and less than high school education were associated with lower risk of non-medical vaccination exemptions. These findings highlight the need to investigate the nuances of groups with different education levels, as they may have attitudinal factors including variations in degrees of vaccination hesitancy, support of compulsory vaccination policies, and access. For example, a study by Kennedy and co-workers done at the individual level reported that persons with at least some college education were more likely to be opposed to compulsory vaccination (Kennedy, Brown & Gust, 2005). While the study by Kennedy, Brown & Gust (2005) focused on individual attitudes towards vaccination, it is interesting to see the contrast between the individual-level findings of their study compared to the county-level associations identified here.

Although this study did not identify higher median income as a predictor of higher non-medical vaccination exemptions, other studies have reported associations between the two. For instance, McNutt et al. (2016) reported that affluence was associated with higher rates of non-medical exemptions in California. Other studies, including the analysis by Yang et al. (2016) of the predictors of vaccination exemptions in California, have reported an association between higher median income and higher percentages of students with non-medical exemptions.

Density of primary care providers was a predictor of higher total vaccination exemptions in this study but was not a significant predictor of non-medical exemptions, which has been reported in other studies. The study by Walker & Rea (2016) reported that for every 10% increase in density of pediatricians there was an 11% decrease in philosophical/personal belief vaccination exemptions in California. However, they also reported that a 10% increase in the proportion of family medicine practitioners was associated with a 3.5% increase in philosophical/personal belief vaccination exemptions (Walker & Rea, 2016). Future individual-level studies should take these practitioner type differences into consideration in investigations aimed at improving our understanding of the socioeconomic and demographic predictors of vaccination exemptions.

### Strengths and limitations

This study has shown evidence of county-level geographic disparities in vaccination exemptions and that these disparities are influenced, at least in part, by socioeconomic and demographic factors. However, this study is not without limitations. Exemption data did not include students in home-school programs and therefore this study could not account for the home-schooled population. Additionally, this analysis was exploratory and only included 1 year of exemption data. Future studies should investigate both temporal trends and geographic disparities in vaccination exemptions and should include more years of data. These limitations notwithstanding, the findings of this study are important for guiding and planning public health programs to reduce vaccination exemptions and improve health outcomes for vaccine-preventable diseases.

## Conclusions

There is evidence of county-level geographic disparities in both total and non-medical vaccination exemption risks in Florida. These disparities are explained by differences in county-level socioeconomic and demographic factors. Study findings are important in guiding resource allocation for health planning aimed at improving vaccination rates and reducing incidence of vaccine-preventable diseases.

## Additional Information and Declarations

### Data Availability

The following information was supplied regarding data availability: The study data are available at Florida Health CHARTS: http://www.flhealthcharts.com/charts/default.aspx.
